# Microalbuminuria Measured by three Different Methods, Blood Pressure and Cardiovascular Risk Factors in Elderly Swedish Males

**Published:** 2008-09-02

**Authors:** Gösta Florvall, Samar Basu, Johanna Helmersson, Anders Larsson

**Affiliations:** 1 Section of Clinical Chemistry, Department of Medical Sciences, Uppsala University Hospital, Uppsala, Sweden; 2 Sections of Geriatrics and Clinical Nutrition Research, Department of Public Health and Caring Sciences, Uppsala University Hospital, Uppsala, Sweden

**Keywords:** Age, Cardiovascular disease, C-reactive protein, Hypertension, IL-6, Inflammation, Microalbuminuria

## Abstract

Microalbuminuria is associated with hypertension and is a strong risk factor for subsequent chronic disease, both renal and coronary heart disease (CHD), Presently there are several methods available for measurement of microalbuminuria. The aim of this study was to evaluate if the three different methods gave similar information or if one of the assays were superior to the others. Blood pressure, inflammatory markers and cardiovascular mortality and morbidity were correlated with urine albumin analysed with a point-of-care testing (POCT) instrument, nephelometric determination of albumin and albumin/creatinine ratio in elderly males. The study population consisted of 103 diabetic and 603 nondiabetic males (age 77 years) in a cross-sectional study. We analyzed urine albumin with a HemoCue^®^ Urine Albumin POCT instrument and a ProSpec^®^ nephelometer and albumin/creatinine ratio. There were strong correlations between both systolic and diastolic blood pressure and all three urine albumin methods (p < 0.0001). There were also significant correlations between the different urine albumin measurements and serum amyloid A component, high-sensitivity C-reactive protein and interleukin-6. The three different urine albumin methods studied provided similar information in relation to cardiovascular disease. There was a strong correlation between systolic and diastolic blood pressure and microalbuminuria in both the whole study population and in nondiabetic males emphasizing the role of hypertension in glomerular damage. The good correlation between the studied urine albumin measurements show that all three methods can be used for monitoring urine albumin excretion.

## Introduction

Hypertension is a highly prevalent disorder that affects the majority of adults (Wang et al. 2005). It is also an important risk factor for myocardial infarction and stroke and it constitutes a major source of cardiovascular morbidity and mortality (Stamler et al. 1993) Experimental and physiological studies support an important role for the kidneys in the pathogenesis of essential hypertension (Cowley and Roman, 1996). In animal hypertension models, one of the earliest findings is a defect in renal sodium excretion resulting in elevated glomerular pressures that leads to endothelial dysfunction and hyperfiltration (Kurokawa, 1995). Previous studies have indicated that microalbuminuria may be a feature of hypertension and a marker of kidney damage in patients with hypertension (Winocour et al. 1992; Horner et al. 1996; Cirillo et al. 1998).

Prevention of nephropathy is one of the most important challenges for modern health care. Moderate chronic renal insufficiency is a disorder with increasing prevalence, particularly in the elderly. In the United States, there are approximately 12.5 million individuals with creatinine clearance less than 50 mL/min/1.73m^2^ (Hsu et al. 2000). Mild to moderate chronic renal insufficiency is a risk factor for acute kidney failure and end-stage renal disease. It is thus important to treat these patients early to prevent them from developing severe kidney damage.

There is an increasing demand for urine albumin tests suitable for point-of-care testing (POCT). POCT offers rapid test results, which facilitates the use of the results to motivate the patient to lifestyle changes and increased compliance (Lehman, 2002; Price, 2000; St-Louis, 2001). To achieve good compliance it is essential that the patient and physician work together and that the patient get regular feedback on the treatment results. The feedback is considered to be more effective if the test results are available during the consultation. This requires that the patient either provides blood samples prior to the consultation or that the care unit has a capability to perform rapid testing of relevant markers. This has led to the development of POCT instruments for urine albumin excretion.

The aim of the present study was to correlate hypertension with different markers for microalbuminuria in a group of elderly males. The methods used in the study were HemoCue^®^ urine albumin (POCT instrument), a central laboratory instrument (ProSpec^®^) and albumin/creatinine ratio (ACR).

## Methods

### Study population

This study is a cross-sectional investigation of Swedish men, 77 years of age, which were participants in Uppsala Longitudinal Study of Adult Men (ULSAM) (Helmersson et al. 2004). This health survey to identify risk factors for cardiovascular disease (CVD) started in 1970; when all men born between 1920–1924, and living in Uppsala, were originally invited to participate (aged 50). These men were reinvestigated at age 77. The study was approved by the Ethics Committee at Uppsala University and all participants gave their informed consent prior to blood sampling. All clinical experimentation described in the manuscript was conducted in accordance with the guidelines proposed in the Declaration of Helsinki.

### Sample collection

Twenty-four hour urine was collected without additives. Blood samples were drawn from an antecubital vein in the morning after a 12-hour (overnight) fast. The samples were immediately frozen at −70°C until analysis. The same urine samples were used for all three assays.

### Medical history, medication and diabetes-definition

Medical history and information on medication were obtained by a self-administered questionnaire. A total of 706 urine samples were analysed. The patients completed a questionnaire and had a fasting plasma glucose value. Type 2 diabetes was diagnosed according to the WHO definition: fasting plasma glucose ≥7.0 mmol/L, or intake of oral anti-diabetic drugs alone, or in combination with insulin treatment (n = 103). Control men had plasma glucose <7.0 mmol/L and no anti-diabetic drug (n = 603). Information of a history of CVD (myocardial infarction, ischemic stroke or angina pectoris) was obtained from the Swedish Hospital Discharge Registry. In the study population, 44.3% of the participants had hypertension, 24% had ischemic heart disease (including myocardial infarction), 16% had a history of myocardial infarction, 18% had cerebrovascular diseases (including stroke) and 16% had a history of stroke.

### Urine albumin

Urine albumin was analyzed with HemoCue^®^ urine albumin (HemoCue^®^, Ängelholm, Sweden). CV calculated on duplicate samples was 3.9%. Urine albumin was also measured by nephelometry (Urine albumin, Dade Behring, Deerfield, IL, U.S.A.) using a Behring BN ProSpec^®^ analyzer (Dade Behring). The total analytical imprecision of the method was 3.7% at 9.5 mg/L and 5.0% at 85 mg/L. Urine creatinine were analyzed with a modified kinetic Jaffe reaction on an Architect Ci8200^®^ analyzer (Abbott, Abbot Park, IL, U.S.A.) and reported as S.I. units (μmol/L) and creatinine related urine albumin was calculated from the Prospec^®^ results. The total analytical imprecision of the creatinine method was 4.8% at both 94 and 337 μmol/L. All assays were performed independently without prior knowledge of other patient data.

### Assays of interleukin (IL)-6 and high-sensitivity C-reactive protein (hsCRP) and serum amyloid A component (SAA)

High sensitivity IL-6 was analyzed by an ELISA kit (IL-6 HS, R&D Systems, Minneapolis, MN, U.S.A.). The total CV of the method was 7% and interassay coefficient of variation (CV) was 5%. High sensitivity CRP and SAA measurements were performed by latex enhanced reagent (Dade Behring, Deerfield, IL, U.S.A.) using a Behring BN ProSpec analyzer (Dade Behring). The intraassay CV of the CRP method was 1.4% at both 1.23 mg/L and 5.49 mg/L and the intraassay CV of the SAA method was 5.9% at 12.8 mg/L and 3.2% at 81.7 mg/L.

### Statistical calculations

All urine albumin values below the detection limits (10 mg/L for HemoCue^®^ and 3 mg/L for ProSpec^®^) was assigned the value of the respective detection limit in the statistical analysis. When the ProSpec^®^ urine albumin values were below 3 mg/L, ACR could not be calculated by the laboratory information system. In the statistical analysis these cases was assigned an ACR of 0.3 g/mol creatinine.

All calculations were performed with the statistical software package Stata 6.0 (Stata Corporation, College Station, Texas, U.S.A.). Differences between diabetic patients and controls were tested with Mann-Whitney test, Kruskal Wallis test or partial correlation in multivariate analyses. Associations between continuous variables were tested with Spearman’s rank correlation analysis. P-values <0.05 were regarded as statistically significant throughout the study.

## Results

### Correlation between urine albumin measured with HemoCue^®^ and ProSpec^®^ and ACR

**Figure 1.**
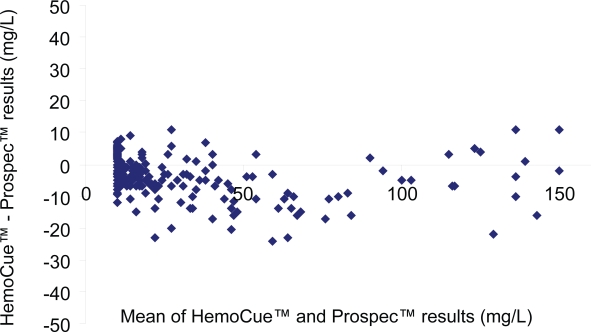
Comparison between urine albumin analyzed with HemoCue® and Prospec® in the range 10–150 mg/L in non-diabetic males. Bland-Altman plot presenting mean of the two measurements (x-axis) versus the difference (HemoCue® - Prospec®) between the two measurements (y-axis).

There was a strong correlation between duplicate analysis of 100 samples on Hemocue^®^ (R^2^ = 0.999). There were also good agreements between the two urine albumin methods (R^2^ = 0.956) (Figure 1) and between ProSpec^®^ and ACR (R^2^ = 0.937) (results not shown). The Hemocue^®^ method gave slightly lower values than ProSpec^®^.

### Prevalence of microalbuminuria in nondiabetic males

114 (18.9%) of the nondiabetic males had urine albumin >20 mg/L and 101 (16.7%) individuals had urine albumin >25 mg/L with the HemoCue^®^ instrument. The corresponding figures for ProSpec^®^ were 124 (20.6%) and 106 (17.6%), respectively. 111 (18.5%) of the nondiabetic males had creatinine related urine albumin >2.5 g/mol creatinine and 102 (17%) individuals had values >3 g/mol creatinine.

### Urine albumin and laboratory markers

No significant correlations were found between any of the microalbuminuria measurements and total cholesterol, HDL-cholesterol or LDL-cholesterol in either the whole study group or the nondiabetic group. In contrast, most of the correlations were significant with triglycerides and the inflammatory markers hsCRP, IL-6 and SAA. The most pronounced correlations were found with SAA. Significant correlations are presented as Spearman rank correlations in Table 1.

### Urine albumin, cardiovascular disease and pharmaceutical treatment

There were significant correlations between urine albumin values and cardiovascular disease and angina but not with ischemic stroke, myocardial infarction and cardiac failure (Table 1). The only significant correlation between urine albumin and pharmaceutical treatment was noted for treatment with calcium antagonists.

## Discussion

The traditional reagent strips for measuring protein-uria are not sensitive enough for monitoring urine albumin concentrations below 100–300 mg/L, thence they do not have sufficient sensitivity to measure microalbuminuria. Microalbuminuria is currently measured by high pressure liquid chromatography (HPLC) and a variety of immunochemical methods, such as radioimmunoassays, enzyme-linked immunoassays, nephelometric and turbidimetric assays (Newman et al. 2005). The instrumentation employed for these assays often limits the use to central laboratories. A few immunoassay-based microalbuminuria tests for point-of-care use have been introduced, such as the Bayer Clinitek 50^®^, DCA 2000^®^ and HemoCue^®^ (Parson et al. 1999a; Parsons et al. 1999b; von Schenck, 2003).

Screening for microalbuminuria are usually performed by one of these methods: measurement of total urine albumin in 12 or 24 h collection, measurement of the albumin-to-creatinine ratio in morning urine or random sample or measurement of urine albumin in morning urine (American Diabetes association 2005). The 24 h collection is time consuming and thus expensive and requires highly motivated patients and careful information and it is often difficult to perform in routine praxis. Urine albumin concentrations are standardised for concurrent creatinine excretion thus obtaining ACR. This procedure is based on the concept that creatinine excretion is stable and corrects for unknown urine volumes. Repeated measurements has shown that albuminuria is twice as variable as creatininuria (Pedrinelli et al. 2001). However, ACR often do not take into account male/female differences and the effect of reduced muscle mass, especially in elderly patients, is usually not adjusted for. Plasma creatinine is also known to be influenced by the diet and the plasma creatinine will influence the urine creatinine. The analytical quality of the creatinine method will influence ACR and there is also an economical aspect of the question, as ACR requires an additional urine creatinine analysis. All these factors should be considered when deciding on which screening method to use.

The lower cut-off values for microalbuminuria varies between studies, but it is often in the range of 15–25 mg/L for albuminuria and 1.8–3 g albumin/mol creatinine for ACR (Bakker, 1999; De Cosmo et al. 2005). The European urinanalysis guideline for ACR is 3.0 g/mol (European confederation of laboratory medicine, 2000), which also is the Swedish laboratory recommendation. The detection limits for both HemoCue^®^ and ProSpec^®^ are sufficient to detect even low levels of microalbuminuria.

ProSpec^®^ gave slightly higher values than HemoCue^®^ (Fig. 1) which also was reflected in a higher number of patients with microalbuminuria when analyzed with ProSpec^®^. However, there was a good agreement between the HemoCue^®^ and ProSpec^®^ quantification of urine albumin especially considering that presently there is an international calibrator for serum but not for urine albumin (Whicher, 1998). Presently, the International Federation of Clinical Chemistry and Laboratory Medicine (IFCC) is planning a work group for preparation of an international calibrator for urine albumin. Such a calibrator is highly needed, as the treatment recommendations for microalbuminuria are international and should not be dependent on the method used. There should also be an international calibrator for urine creatinine for ACR.

We found strong correlations between albuminuria and blood pressure, triglycerides, hsCRP and SAA. There were also significant correlations between urine albumin and cardiovascular disease and angina but not with death due to myocardial infarction, heart failure or stroke. This may be due to the difference in events. There were considerable more patients suffering from cardiovascular disease than the number of patients that died due to myocardial infarction. A longer follow up period may have resulted in more significant correlations with the mortality and morbidity markers. There was a positive correlation between urine albumine excretion and use of calcium antagonists but not with the other antihypertensive drugs. The positive correlation is a bit unexpected, as one of the aims of the antihypertensive treatment is to reduce albuminuria. However, this may be due to a treatment bias as the choice of drugs may be influenced by the albuminuria.

In conclusion, this study shows a good correlation between urine albumin analysed with HemoCue^®^ POCT instrument, a central laboratory instrument (ProSpec^®^) and ACR in a group of elderly males showing that all three methods can be used for monitoring urine albumin excretion. Age, sex and ethnicity composition were homogenous in this study, but the results may therefore have limited generalizability to other age and ethnic groups and women. The strong correlation between systolic and diastolic blood pressure and microalbuminuria in both the whole population and in nondiabetic males shows that the blood pressure has an important role for the development of albuminuria.

## Figures and Tables

**Table 1.** Spearman Rank correlations between urine albumin values and blood pressure, laboratory markers, medication and cardiovascular disease. Only correlations with a p value <0.05 for the whole population is presented.

	**All males**	**Nondiabetic males**
	r	r
**Systolic blood pressure**		
U-albumin, HemoCue^®^	0.237	0.234
U-albumin, ProSpec^®^	0.269	0.272
ACR	0.267	0.272
**Diastolic blood pressure**		
U-albumin, HemoCue^®^	0.187	0.176
U-albumin, ProSpec^®^	0.211	0.210
ACR	0.213	0.207
**Triglycerides**		
U-albumin, HemoCue^®^	0.106	NS
U-albumin, ProSpec^®^	0.140	0.129
ACR	0.131	0.108
**HsCRP**		
U-albumin, HemoCue^®^	0.119	0.128
U-albumin, ProSpec^®^	0.119	0.105
ACR	0.101	NS
**SAA**		
U-albumin, HemoCue^®^	0.163	0.165
U-albumin, ProSpec^®^	0.170	0.144
ACR	0.183	0.152
**IL-6**		
U-albumin, HemoCue^®^	0.150	0.125
U-albumin, ProSpec^®^	0.118	0.085
ACR	0.112	NS
**Angina**		
U-albumin, HemoCue^®^	0.112	0.120
U-albumin, ProSpec^®^	0.120	0.133
ACR	0.137	0.138
**Cardiovascular disease**		
U-albumin, HemoCue^®^	0.123	0.101
U-albumin, ProSpec^®^	0.093	NS
ACR	0.113	0.084
**Calcium antagonists**		
U-albumin, HemoCue^®^	0.144	0.143
U-albumin, ProSpec^®^	0.134	0.141
ACR	0.147	0.151
